# Is pain relief enough? Early response discordance after lumbar decompressive surgery: a prospective cohort study

**DOI:** 10.1186/s12891-026-10195-3

**Published:** 2026-07-10

**Authors:** Tomasz Szczepański, Marta Koźba-Gosztyła, Anastasija Krzemińska, Grzegorz Miękisiak, Bogdan Czapiga

**Affiliations:** 1 Clinical Department of Neurosurgery, Center for Neurologic Disorders, 4th Military Hospital, Wroclaw, Poland; 2 Vratislavia Medica Research Institute, Wrocław, Poland; 3 Department of Clinical Neurosciences, Wrocław University of Science and Technology ­, Wrocław, Poland

**Keywords:** Lumbar decompression, Patient-reported outcome measures, Minimal clinically important difference, Response discordance, Oswestry Disability Index, EQ-5D-5L, Visual analogue scale, Spinal stenosis, Disc herniation, Outcome assessment

## Abstract

**Background:**

Different patient-reported outcome measures (PROMs) may classify the same patient differently as a treatment responder after lumbar decompressive surgery. We aimed to quantify inter-instrument agreement on minimal clinically important difference (MCID)-based responder status at one month postoperatively, characterize multi-dimensional response discordance, and identify independent one-month PROM predictors of one-month patient dissatisfaction.

**Methods:**

In this prospective observational cohort study, 158 consecutive adults undergoing lumbar decompressive surgery for disc herniation (*n* = 77) or spinal stenosis (*n* = 81) at a single neurosurgical centre were enrolled; all underwent single-level decompression of the symptomatic level. Of these, 134 (84.8%) completed paired baseline and one-month assessments using the Visual Analogue Scale for leg pain (VAS), Oswestry Disability Index (ODI), and EQ-5D-5 L utility index. MCID thresholds were derived from external sources (VAS ≥ 1.5 points, ODI ≥ 10 points, EQ-5D-5 L ≥ 0.10). Inter-instrument agreement was quantified using Cohen’s kappa (κ). Patient dissatisfaction was defined by the satisfaction subscale of the Swiss Spinal Stenosis Questionnaire (SSSQ > 12). Independent predictors of dissatisfaction were identified by multivariable linear regression with bootstrapped confidence intervals.

**Results:**

All instruments showed significant improvement at one month (all *p* < 0.001). MCID-based responder rates differed markedly: VAS 82.1%, EQ-5D-5 L 83.6%, and ODI 54.5%. Inter-instrument agreement was slight to fair (κ = 0.19–0.29); Only 47.0% of patients achieved MCID across all three instruments simultaneously, while 94.0% achieved it on at least one — producing a 47-percentage-point disagreement zone. A total of 39.6% (53/134) of patients experienced VAS and/or EQ-5D-5 L improvement without corresponding ODI improvement. 23% (31/134) of patients were dissatisfied at one month. In multivariable regression adjusted for age, sex, and diagnosis (R² = 0.40), one-month EQ-5D-5 L items of mobility limitation and anxiety/depression, together with residual functional disability (ODI total), independently predicted dissatisfaction; residual pain did not.

**Conclusions:**

Instrument selection alone changes responder classification rates by nearly 30% points at one month after lumbar decompressive surgery. Dissatisfaction is driven by residual functional limitation and psychological distress, not residual pain. Multi-dimensional PROM assessment with diagnosis-informed instrument selection may help identify, at the early postoperative visit, patients requiring targeted rehabilitation and psychological support.

**Trial registration:**

Not applicable.

**Supplementary Information:**

The online version contains supplementary material available at https://doi.org/10.1186/s12891-026-10195-3.

## Background

Patient-reported outcome measures (PROMs) are the standard for evaluating surgical benefit in lumbar degenerative spine surgery. Instruments such as the Visual Analogue Scale (VAS) for pain, the Oswestry Disability Index (ODI) for function, and the EQ-5D-5 L for health-related quality of life each capture a distinct outcome domain. These instruments are used to determine whether a patient has achieved a clinically meaningful improvement, typically by applying a minimal clinically important difference (MCID) threshold. A commonly held but largely untested assumption underlying this practice is that different instruments classify the same patients as treatment responders — that a patient who improves in pain also improves in function and quality of life [1].

This assumption has been challenged. Wertli et al. [2] demonstrated in a cohort of 466 patients with lumbar spinal stenosis that the prevalence of patients fulfilling MCID criteria ranged from 40% to 70% depending on the instrument, with inter-instrument agreement of only κ = 0.24–0.50 (fair to moderate). Mannion et al. [3] reported 22% individual-level disagreement between ODI and COMI responder status (κ = 0.56) in 3 324 patients. These findings suggest that pain, function, and quality of life may represent distinct recovery trajectories rather than a single unitary outcome. However, no study has systematically characterised the pattern, directionality, and clinical predictors of this inter-instrument discordance, nor has any study compared discordance patterns between disc herniation and spinal stenosis.

Recovery after lumbar decompressive surgery is a multi-dimensional process that unfolds over 12 to 24 months, but the rate of improvement differs substantially across outcome domains. The Spine Patient Outcomes Research Trial (SPORT) demonstrated that surgical patients with disc herniation achieved approximately 96% of their eventual 8-year pain improvement (SF-36 Bodily Pain) within the first 3 months, with little further gain thereafter [4, 5]; for spinal stenosis, a significant surgical advantage emerged by 3 months and was maintained at 2 years [6]. The Spine Tango registry (*n* = 4 287) confirmed this temporal pattern at finer resolution: the dominant recovery phase occurred from preoperatively to 3 months (COMI change 3.6 ± 2.8 points), with only a small additional gain from 3 to 12 months (0.3 ± 2.4 points) and no further significant change from 12 to 60 months [7]. The Swedish Spine Register equivalence study (*n* ≈ 98 700) corroborated that the proportion of patients reaching minimal important change on VAS, ODI, and EQ-5D differed by less than 2% between 1- and 2-year follow-up, establishing that PROMs stabilise after 12 months [8]. Critically, however, these aggregate trajectories mask a fundamental domain-specific asymmetry. DeVine et al. [9] showed in a systematic review that pooled effect sizes after lumbar surgery were large for both pain (VAS: 1.4 ± 0.57) and function (ODI: 1.1 ± 0.39), with overlapping confidence intervals, and medium for quality of life (EQ-5D: 0.78 ± 0.12), with no inter-domain correlation exceeding ρ = 0.70 — confirming that these instruments measure genuinely distinct constructs that recover at different rates. This differential responsiveness translates into measurable clinical discordance at early timepoints: Asher et al. [10] reported in the N2QOD registry (*n* = 3 073) that 23% of patients were discordant for MCID achievement on the ODI between 3 and 12 months, and that substantial clinical benefit rose from 56% at 3 months to 61% at 12 months, while MCID achievement rose only from 68% to 71%, representing continued functional improvement well after pain relief had plateaued. Systematic-review evidence on the early clinical course of pain and disability after lumbar discectomy similarly indicates that disability continues to improve over the first postoperative months [11].

These converging lines of evidence indicate that the first postoperative clinical contact — typically at 4 to 6 weeks — occupies a unique position in the recovery continuum: the acute surgical effects have resolved, the majority of pain improvement has already occurred [4, 5, 7], but functional reconditioning and psychological adaptation are still unfolding [10]. This creates the biological and psychometric conditions under which different PROMs may yield discordant classifications of the same patient, and the clinical conditions under which such discordance, if identified, remains modifiable through targeted rehabilitation and psychological support [12, 13]. Understanding whether and how this asymmetry manifests as measurable inter-instrument discordance at the first postoperative visit is therefore essential for directing early care — yet no study has systematically characterised these patterns at this timepoint.

We therefore aimed to: (1) quantify the inter-instrument agreement between pain (VAS), function (ODI), and quality of life (EQ-5D-5 L index) on MCID-based responder status at 1 month after lumbar degenerative spine surgery; (2) determine whether discordance patterns differ between disc herniation and spinal stenosis; and (3) identify independent one-month PROM predictors of one-month patient dissatisfaction that should be prioritised in early postoperative rehabilitation.

## Materials

### Study design and setting

This work represents a secondary analysis of data from the PROGRES study (*Evaluation of the natural course of lumbar degenerative spine disease and factors influencing its progression*,* with an analysis of the impact of surgical intervention as a modifying factor in the long-term course of the disease – prospective observational study*), an ongoing prospective observational cohort study conducted at the Neurosurgical Department of the 4th Military Clinical Hospital in Wrocław, Poland. The PROGRES study employs a cohort design to evaluate the natural history and clinical outcomes of lumbar degenerative disease before and after surgical treatment. The present analysis uses preoperative assessment data and 1-month postoperative follow-up data; patients were enrolled between May and December 2025, and one-month follow-up was completed by January 2026.

The study is purely observational, involving the analysis of clinical, imaging, and patient-reported outcome data without any intervention in the diagnostic or therapeutic process. The research protocol was developed in accordance with the Strengthening the Reporting of Observational Studies in Epidemiology (STROBE) guidelines for cohort studies (Supplementary checklist).

### Participants

Consecutive adult patients hospitalised at the Neurosurgical Department with a confirmed diagnosis of lumbar degenerative disease were screened for eligibility. Lumbar degenerative disease encompassed two primary diagnoses: lumbar spinal stenosis and lumbar disc herniation. Radiographically, the stenosis group comprised patients with central canal stenosis of Schizas grade B–D on magnetic resonance imaging, and the disc-herniation group comprised patients with a disc-related radicular conflict without spinal canal stenosis.

Inclusion criteria were as follows: age between 18 and 90 years, clinically relevant degenerative changes of the lumbar spine confirmed by magnetic resonance imaging (MRI), ability to participate in long-term follow-up assessments, provision of written informed consent, and cognitive status sufficient to understand the purpose and methods of the study and complete questionnaires.

Exclusion criteria included the following: history of previous spinal surgery; spinal neoplasms; neurological or muscular diseases causing motor function impairment (e.g., amyotrophic lateral sclerosis, multiple sclerosis, or myopathies); and inability to participate in long-term observation due to severe chronic illness with limited life expectancy. No participant in the studied cohort had a known psychiatric illness.

Between May and December 2025, determination of eligibility was conducted by a trained team comprising spine surgeons and clinical research coordinators. Following clinical assessment, all eligible patients completed standardized questionnaires on the day of hospital admission, prior to the consultation during which the surgical treatment plan and operative approach were discussed. All patients subsequently underwent single-level decompression of the symptomatic level. Decompression was performed unilaterally with an over-the-top approach or bilaterally, individualised to the clinical presentation and imaging findings by the operating surgeon, with or without discectomy; no instrumented procedures and no revision surgery were included in the analysed cohort. All patients were informed about the study process and methods and about their right to withdraw at any time.

### Ethics

The study protocol was approved by the Bioethics Committee of the Lower Silesian Medical Chamber in Wrocław (approval number: 09/DOBD/2025, date: April 9, 2025). The study was conducted in accordance with the tenets of the Declaration of Helsinki and its subsequent amendments. All participants provided written informed consent prior to enrolment. Participants were informed of their right to withdraw from the study at any time without consequences for their medical care. Data were anonymised and stored securely in compliance with applicable data protection regulations.

### Outcome measures

PROMs were collected at baseline (preoperative, on the day of hospital admission) and at 1 month postoperatively. Pain intensity was assessed using VAS for leg pain and back pain (0–10 scale). Functional disability was measured using the Polish validated version of the Oswestry Disability Index (ODI; 0–100 scale) [14]. Health-related quality of life was assessed with the EQ-5D-5 L, converted to a utility index using the Polish value set (Golicki et al. [15]). Patient satisfaction was assessed at follow-up using the satisfaction subscale of the Swiss Spinal Stenosis Questionnaire (SSSQ) as an external criterion independent of the PROMs under study; dissatisfaction was defined as SSSQ > 12 [16]. One-month assessments were administered by an external assessor blinded to the surgical diagnosis, before the scheduled surgical consultation.

### MCID thresholds and responder definition

MCID thresholds were derived from published external literature and were not estimated within this cohort, thereby avoiding circularity with the SSSQ-based satisfaction criterion. The primary analysis used moderate thresholds: VAS leg pain ≥ 1.5 points [17], ODI ≥ 10 points [18], and EQ-5D-5 L index ≥ 0.10 [19]. These thresholds are consistent with pathology-specific estimates from the Canadian Spine Outcomes and Research Network (CSORN) registry, in which Power et al. [20] reported MCID values for ODI change ranging from − 10.0 to − 16.9 depending on lumbar pathology, supporting our use of the moderate ≥ 10-point threshold; this value also falls centrally within the 4–20-point ODI MCID range reported across spine-surgery studies in a recent systematic review [18]. Sensitivity analyses were conducted at liberal (VAS ≥ 1.0, ODI ≥ 6, EQ-5D-5 L ≥ 0.05) and conservative (VAS ≥ 2.0, ODI ≥ 14, EQ-5D-5 L ≥ 0.15) thresholds; the slight-to-fair agreement and the disagreement zone were preserved across all threshold sets.

### Statistical analysis

Baseline characteristics were compared between diagnostic groups using Mann–Whitney U tests and chi-square tests. The use of nonparametric tests was supported by Shapiro–Wilk testing and inspection of distributional plots, which indicated departures from normality and floor/ceiling effects for several PROMs. Within-group change was assessed by Wilcoxon signed-rank test; effect sizes are reported as Cohen’s d.

Inter-instrument agreement on MCID-based responder status was quantified using Cohen’s kappa (κ), supplemented by overall percent agreement, specific positive agreement (Ppos), and specific negative agreement (Pneg) to address the known prevalence paradox of κ [21]. Kappa was interpreted according to Landis and Koch (1977): <0.20 slight, 0.21–0.40 fair, 0.41–0.60 moderate [22]. Multi-dimensional response discordance was defined as achieving MCID in pain and/or quality of life but not in function (Type A discordance) or in function but not in pain/quality of life (Type B discordance).

Criterion validity of each PROM against SSSQ satisfaction was assessed via Spearman correlations and ROC analysis with 2 000-iteration bootstrap 95% confidence intervals. Independent predictors of dissatisfaction were identified by multivariable linear regression with the continuous SSSQ satisfaction score as the dependent variable. Candidate predictors were prespecified on clinical grounds: one-month VAS leg pain, VAS back pain, and ODI total score, together with the EQ-5D-5 L mobility and anxiety/depression items; the model was adjusted for age, sex, and primary diagnosis. Individual ODI subscale items were not entered, the reliable ODI total score being used instead. With 134 observations and eight model terms (approximately 17 observations per term), the model was adequately specified for linear regression; collinearity was assessed by variance inflation factors and stability by bootstrapped 95% confidence intervals. The analysis remains exploratory pending external validation. Analyses were performed in Python 3.11 (SciPy, scikit-learn). Statistical significance was set at *p* < 0.05.

## Results

### Patient flow and baseline characteristics

Of 161 patients screened, 3 were excluded (did not undergo surgery). Of 158 operated patients (disc herniation *n* = 77; spinal stenosis *n* = 81), 134 (84.8%) completed the 1-month follow-up with full paired data; 24 (15.2%) had incomplete follow-up. Lost patients did not differ in age (56.8 ± 16.0 vs. 60.2 ± 14.5; *p* = 0.259) or baseline VAS leg (6.8 vs. 6.7; *p* = 0.692), though baseline ODI was marginally higher (32.7 vs. 29.7; *p* = 0.035).

The analysed cohort (*n* = 134) had a mean age of 60.2 ± 14.5 years (range 27–81), 59.7% were female. Stenosis patients were significantly older than disc herniation patients (66.7 ± 9.6 vs. 51.9 ± 15.5; *p* < 0.001); sex, education, and all baseline PROMs did not differ between diagnoses (all *p* > 0.18) (Table 1).

**Table 1:** Baseline characteristics of the study cohort stratified by primary diagnosis

Variable	Disc herniation (n = 59)	Spinal stenosis (n = 75)	Total (n = 134)	p-value
Demographics				
Age, years; mean ± SD (range)	51.9 ± 15.5 (27–80)	66.7 ± 9.6 (41–81)	60.2 ± 14.5 (27–81)	< 0.001 a
Sex, female; n (%)	33 (55.9%)	47 (62.7%)	80 (59.7%)	0.541 b
Education level; n (%)				
None	5 (8.5%)	0 (0.0%)	5 (3.7%)	
Primary	2 (3.4%)	3 (4.0%)	5 (3.7%)	
Vocational	12 (20.3%)	17 (22.7%)	29 (21.6%)	
Secondary	24 (40.7%)	36 (48.0%)	60 (44.8%)	
Higher	16 (27.1%)	19 (25.3%)	35 (26.1%)	0.141 b
Baseline patient-reported outcome measures; mean ± SD				
VAS Leg Pain (0–10)	6.8 ± 2.4	6.7 ± 2.9	6.7 ± 2.7	0.831 a
VAS Back Pain (0–10)	5.7 ± 2.9	5.8 ± 3.0	5.8 ± 2.9	0.780 a
ODI total (0–100)	30.5 ± 7.9	29.1 ± 6.6	29.7 ± 7.2	0.355 a
EQ-5D-5L index (−0.59–1.00) c	0.42 ± 0.28	0.42 ± 0.27	0.42 ± 0.28	0.886 a

*SD* standard deviation, *VAS* Visual Analogue Scale, *ODI* Oswestry Disability Index, *EQ-5D-5L* EuroQol five-dimension five-level instrument

Significance threshold: *p* < 0.05. Values in bold indicate statistically significant differences

aMann–Whitney U test

bChi-square test

cEQ-5D-5L utility index computed using the Polish population-based value set (Golicki et al., PharmacoEconomics 2019)

Table 1. Baseline characteristics of the study cohort stratified by primary diagnosis. Data are presented as mean ± standard deviation for continuous variables and as number (percentage) for categorical variables. p-values refer to between-diagnosis comparisons (Mann–Whitney U test for continuous variables; chi-square test for categorical variables). The EQ-5D-5 L utility index was computed using the Polish population-based value set (Golicki et al., PharmacoEconomics 2019). SD = standard deviation; VAS = Visual Analogue Scale; ODI = Oswestry Disability Index.

### Early postoperative improvement

All instruments showed significant improvement at 1 month (all *p* < 0.001). VAS leg pain decreased from 6.7 ± 2.7 to 2.0 ± 2.6 (mean change 4.7 ± 3.5; Cohen’s d = 1.36; 70% relative improvement), EQ-5D-5 L index increased from 0.42 ± 0.28 to 0.80 ± 0.16 (Δ = 0.38 ± 0.29; d = 1.30), and ODI decreased from 29.7 ± 7.2 to 19.9 ± 6.5 (Δ = 9.8 ± 9.6; d = 1.02; 33% relative improvement). Recovery was asymmetric across instruments: relative improvement and MCID attainment were higher for pain and quality of life than for function. Because the instruments differ in measurement properties, including ODI floor and ceiling effects, these magnitudes are not directly comparable across scales.

MCID-based responder rates at moderate thresholds were: VAS leg 82.1%, EQ-5D-5 L 83.6%, and ODI 54.5%. Responder rates for pain and quality of life were thus approximately 30% points higher than for function.

### Inter-instrument agreement

**Fig. 1:**
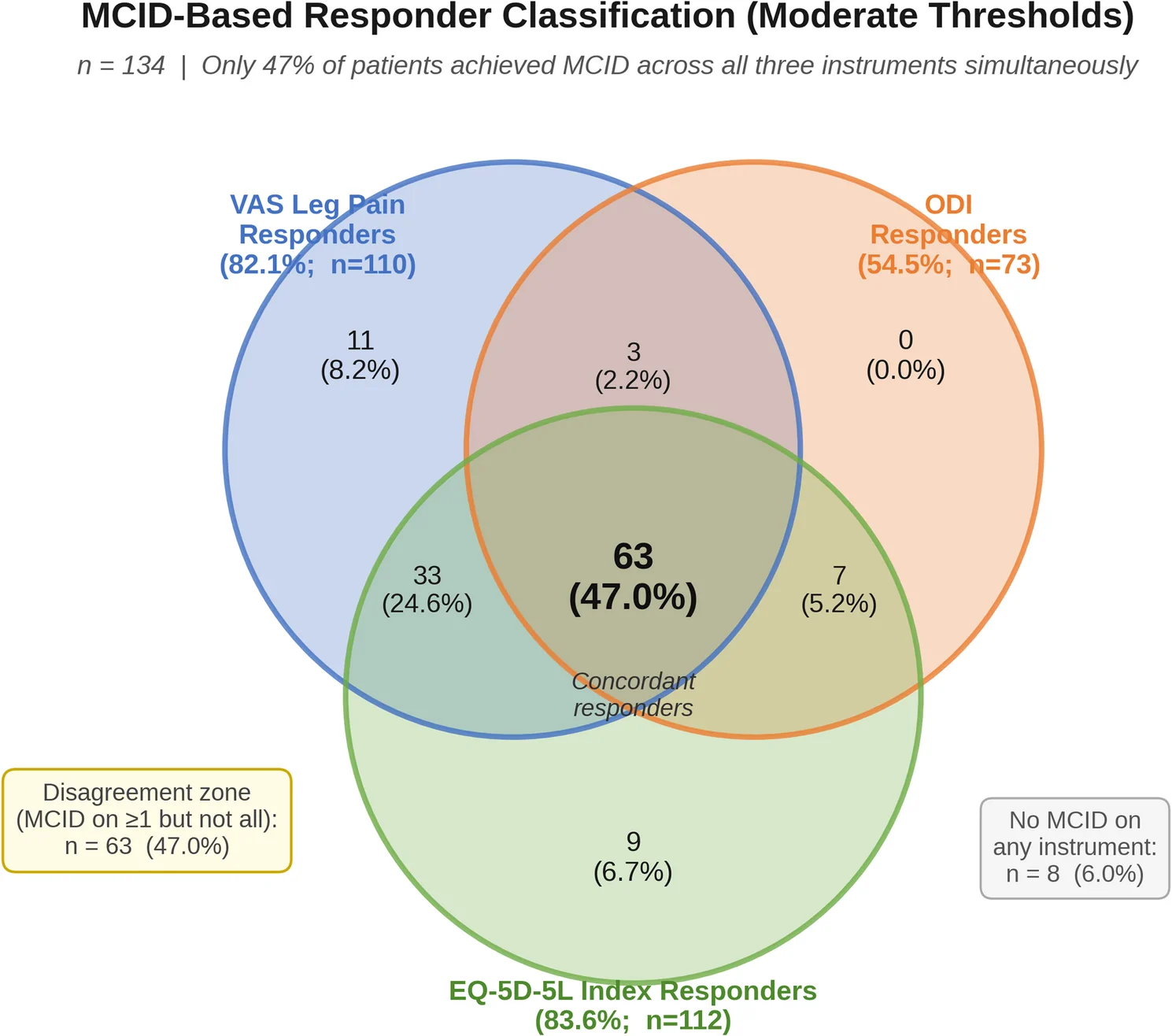
Venn diagram of MCID-based responder classification at 1-month follow-up (moderate thresholds; *n* = 134). Circles represent patients achieving MCID on VAS Leg Pain (≥ 1.5 pts; 82.1%), ODI (≥ 10 pts; 54.5%), and EQ-5D-5 L index (≥ 0.10; 83.6%). The central overlap denotes concordant responders across all three instruments (*n* = 63; 47.0%); an equal proportion fell into the disagreement zone (*n* = 63; 47.0%). Eight patients (6.0%) achieved MCID on no instrument. Inter-instrument agreement: Cohen’s κ = 0.19–0.29

Cohen’s κ for pairwise agreement on responder status ranged from 0.19 to 0.29, indicating slight to fair agreement (Table 2). Overall percent agreement ranged from 61.9% (VAS vs. ODI) to 77.6% (VAS vs. EQ-5D-5 L), but the high agreement for VAS vs. EQ-5D-5 L largely reflected both instruments classifying patients as responders (Ppos = 0.87), with poor agreement on non-responders (Pneg = 0.35). Only 47.0% of patients achieved MCID across all three instruments simultaneously, while 94.0% achieved it on at least one — producing a 47-percentage-point disagreement zone (Fig. 1). This pattern was stable across all MCID threshold levels (κ range 0.15–0.31; disagreement zone 33.6–53.0%).

**Table 2:** Inter-instrument agreement on MCID-based responder status (moderate thresholds)

Statistic	VAS Leg Pain vs. ODI	VAS Leg Pain vs. EQ-5D-5 L	ODI vs. EQ-5D-5 L
Responder rates (MCID achieved); *n* (%)			
Instrument A responders	110 (82.1%)	110 (82.1%)	73 (54.5%)
Instrument B responders	73 (54.5%)	112 (83.6%)	112 (83.6%)
**2 × 2 responder classification matrix; n**			
Both responders (R / R)	**66**	**96**	**70**
Instrument A responder only (R / NR)	44	14	3
Instrument B responder only (NR / R)	7	16	42
Neither responder (NR / NR)	**17**	**8**	**19**
**Agreement statistics**			
Overall percent agreement (%)	61.9%	77.6%	66.4%
Cohen’s κ [95% CI] a	**0.19 [0.06–0.32]**	**0.21 [0.08–0.34]**	**0.29 [0.15–0.42]**
Interpretation b	Slight	Slight	Fair
Specific agreement indices (addressing prevalence paradox) c			
Specific positive agreement (Ppos)	0.72	0.87	0.76
Specific negative agreement (Pneg)	0.40	0.35	0.46
Prevalence index (PI)	0.24	0.55	0.33
**Multi-dimensional concordance across all three instruments**			
Concordant responders (MCID on all three); n (%)	**63 (47.0%)**	**—**	**—**
Disagreement zone (MCID on ≥ 1 but not all); n (%)	**63 (47.0%)**	**—**	**—**
No MCID on any instrument; n (%)	8 (6.0%)	—	—
Inter-instrument disagreement range (pp) d	29.1 pp	—	—

*MCID* minimal clinically important difference, *VAS* Visual Analogue Scale (leg pain), *ODI* Oswestry Disability Index, *EQ-5D-5 L* EuroQol five-dimension five-level utility index, *pp* percentage points, *R* responder, *NR* non-responder, *PI* prevalence index, *Ppos* specific positive agreement, *Pneg* specific negative agreement

a 95% confidence intervals estimated by bootstrap (2 000 iterations)

b Interpreted according to Landis & Koch (1977): κ < 0.20 = slight; 0.21–0.40 = fair

c Ppos = 2a / (2a + b + c); Pneg = 2d / (2d + b + c); PI = (a + d − b − c) / n. Reported to address the known prevalence paradox of κ (Feinstein & Cicchetti, 1990)

d Difference between the highest (EQ-5D-5 L, 83.6%) and lowest (ODI, 54.5%) responder rates. Pairwise columns for the multi-dimensional concordance rows apply to the overall three-instrument analysis and are therefore not disaggregated by pair (—)

MCID thresholds (moderate): VAS Leg Pain ≥ 1.5 points; ODI ≥ 10 points; EQ-5D-5 L index ≥ 0.10. *n* = 134

Table 2. Inter-instrument agreement on MCID-based responder status at 1-month follow-up (moderate thresholds; *n* = 134). Cohen’s κ interpreted per Landis & Koch (1977). Ppos, Pneg, and PI reported to address the prevalence paradox of κ. MCID thresholds: VAS Leg Pain ≥ 1.5 pts; ODI ≥ 10 pts; EQ-5D-5 L index ≥ 0.10. CI = confidence interval; NR = non-responder; ODI = Oswestry Disability Index; R = responder; VAS = Visual Analogue Scale.

### Multi-dimensional response discordance

Pain/quality-of-life versus function discordance occurred in 39.6% of patients, exclusively as Type A: pain and/or QoL improved while function did not. The reverse pattern (Type B) did not occur in any patient. Type A discordance was numerically more prevalent in spinal stenosis (45.3%) than disc herniation (32.2%), though this did not reach statistical significance (OR = 1.75; *p* = 0.155).

### Dissatisfied patient profile

Thirty-one patients (23.1%) were dissatisfied. They were significantly older (66.7 ± 12.0 vs. 58.2 ± 14.6 years; *p* = 0.002), but baseline PROM scores were identical to those of satisfied patients across all instruments (all *p* > 0.19; Table 2). Dissatisfaction arose from insufficient improvement rather than worse initial status: VAS leg improved by 49% in dissatisfied vs. 76% in satisfied patients, EQ-5D-5 L index Δ = 0.32 vs. 0.42, and ODI by only 17% vs. 38%. The asymmetry was most pronounced for function.

**Table 3:** Baseline and postoperative characteristics of satisfied and dissatisfied patients

Variable	Satisfied (*n* = 103)	Dissatisfied (*n* = 31)	*p*-value
Demographics			
Age, years; mean ± SD	58.2 ± 14.6	66.7 ± 12.0	**0.002 a**
Sex, female; n (%)	64 (62.1%)	16 (51.6%)	0.494 b
Spinal stenosis; n (%)	56 (54.4%)	19 (61.3%)	0.880 b
SSSQ score; mean ± SD	8.8 ± 2.2	14.2 ± 1.7	**< 0.001 a**
**Baseline patient-reported outcome measures; mean ± SD**			
VAS Leg Pain (0–10)	6.9 ± 2.4	6.1 ± 3.3	0.309 a
VAS Back Pain (0–10)	5.8 ± 2.9	5.6 ± 3.0	0.826 a
ODI total (0–100)	29.9 ± 7.2	29.4 ± 7.3	0.796 a
EQ-5D-5 L index score	0.45 ± 0.26	0.38 ± 0.32	0.305 a
**1-month follow-up patient-reported outcome measures; mean ± SD**			
VAS Leg Pain (0–10)	1.7 ± 2.4	3.1 ± 3.2	**0.015 a**
VAS Back Pain (0–10)	2.0 ± 2.3	3.3 ± 3.1	0.060 a
ODI total (0–100)	18.6 ± 6.0	24.4 ± 5.8	**< 0.001 a**
EQ-5D-5 L index score	0.87 ± 0.12	0.70 ± 0.21	**< 0.001 a**
**Change scores (baseline → 1 month); mean ± SD**			
ΔVAS Leg Pain	5.3 ± 3.3	3.0 ± 3.7	**0.002 a**
ΔVAS Back Pain	3.7 ± 3.5	2.4 ± 3.6	0.059 a
ΔODI (decrease = improvement)	11.3 ± 9.3	5.0 ± 9.0	**0.001 a**
ΔEQ-5D-5 L index score (increase = improvement)	+ 0.42 ± 0.28	+ 0.32 ± 0.35	0.065 a
Relative improvement — VAS Leg Pain (%)	75.4%	49.2%	— c
Relative improvement — ODI (%)	37.8%	17.0%	— c
**MCID-based responder rates at 1 month; n (%) d**			
VAS Leg Pain responders (≥ 1.5 pts)	90 (87.4%)	20 (64.5%)	**0.005 b**
ODI responders (≥ 10 pts)	63 (61.2%)	10 (32.3%)	**0.006 b**
EQ-5D-5 L responders (≥ 0.10 index) e	90 (87.4%)	22 (71.0%)	0.031 b
**Concordant responders (VAS + ODI); n (%)**	**57 (55.3%)**	**9 (29.0%)**	**0.009 b**
**Residual symptom profile at 1 month in dissatisfied patients; n (%)**			
No residual leg pain (VAS = 0)	—	12 (38.7%)	—
Moderate functional disability (ODI 21–40)	—	22 (71.0%)	—
EQ-5D-5 L: mobility problems (score > 1)	—	18 (58.1%)	—
EQ-5D-5 L: depression / anxiety (score > 1)	—	19 (61.3%)	—

Dissatisfaction defined as SSSQ satisfaction subscale score > 12. Significant p-values (*p* < 0.05) are shown in red. *MCID* minimal clinically important difference, *ODI* Oswestry Disability Index, *SD* standard deviation, *SSSQ* Swiss Spinal Stenosis Questionnaire, *VAS* Visual Analogue Scale

a Mann–Whitney U test

b Chi-square test

c Relative improvement not tested statistically due to zero baseline values in a subset of patients

d MCID thresholds (moderate): VAS Leg Pain ≥ 1.5 pts; ODI ≥ 10 pts; EQ-5D-5 L utility index ≥ 0.10

e EQ-5D-5 L responder rate estimated from utility index change; computed using the Polish value set (Golicki et al., 2019)

— Not applicable (statistic reported for dissatisfied group only or not calculated)

Table 2. Baseline and postoperative characteristics of satisfied and dissatisfied patients at 1-month follow-up. Dissatisfaction defined as SSSQ satisfaction subscale score > 12. Data are presented as mean ± SD for continuous variables and as n (%) for categorical variables. Significant p-values (*p* < 0.05) are highlighted. MCID thresholds (moderate): VAS Leg Pain ≥ 1.5 pts; ODI ≥ 10 pts; EQ-5D-5 L index ≥ 0.10. MCID = minimal clinically important difference; ODI = Oswestry Disability Index; SD = standard deviation; SSSQ = Swiss Spinal Stenosis Questionnaire; VAS = Visual Analogue Scale.

MCID-based responder rates in dissatisfied patients were: VAS leg 64.5%, EQ-5D-5 L 71.0%, but ODI only 32.3%. Concordant response on both VAS and ODI was achieved by 29.0% of dissatisfied vs. 55.3% of satisfied patients.

The residual symptom profile of dissatisfied patients was notable: 38.7% reported no leg pain (VAS = 0) at follow-up, yet 71.0% maintained moderate functional disability (ODI 21–40). At the EQ-5D-5 L dimension level, 77.4% still reported pain/discomfort, 61.3% depression/anxiety, and 58.1% mobility problems.

### Independent predictors of dissatisfaction

**Fig. 2:**
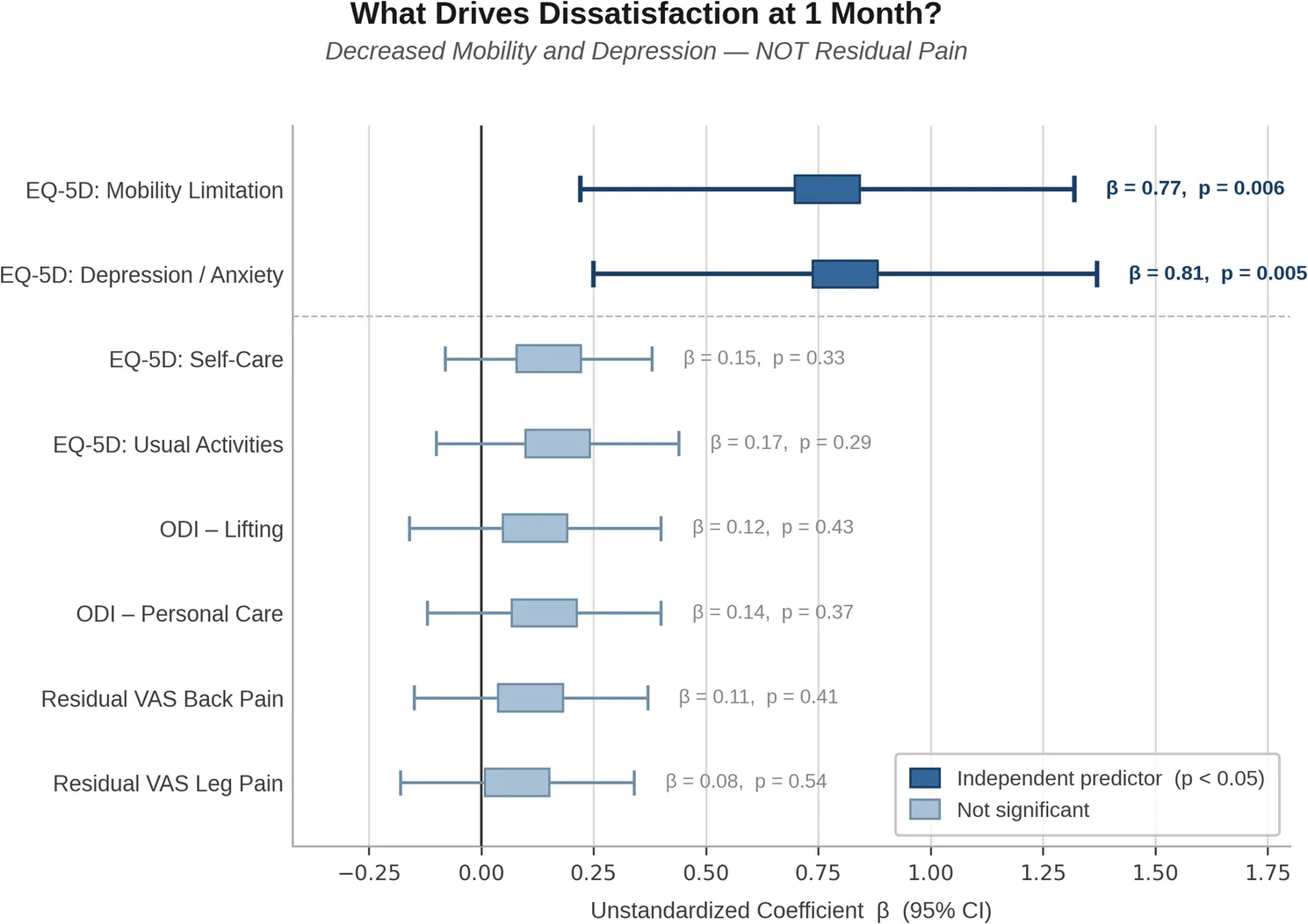
Coefficient plot from multivariable linear regression (dependent variable: SSSQ satisfaction score; *n* = 134). Filled squares represent unstandardized regression coefficients (β); horizontal lines indicate 95% bootstrapped confidence intervals. Independent predictors of dissatisfaction were residual mobility limitation (β = 0.62; 95% CI 0.09–1.16; *p* = 0.023), anxiety/depression (β = 0.80; 95% CI 0.24–1.35; *p* = 0.005), and residual functional disability (ODI total; β = 0.14; 95% CI 0.06–0.22; *p* = 0.001); residual leg and back pain (VAS) were non-significant. The model was adjusted for age, sex, and diagnosis. The dashed horizontal line separates significant from non-significant predictors. Model R² = 0.40

In multivariable linear regression adjusted for age, sex, and primary diagnosis (R² = 0.40), three one-month variables independently predicted dissatisfaction: residual mobility limitation (β = 0.62; 95% CI 0.09–1.16; *p* = 0.023), anxiety/depression (β = 0.80; 95% CI 0.24–1.35; *p* = 0.005), and residual functional disability (ODI total; β = 0.14; 95% CI 0.06–0.22; *p* = 0.001). Residual VAS leg and back pain were not significant after adjustment; female sex was independently associated with greater satisfaction (β = −1.00; 95% CI − 1.90 to − 0.10; *p* = 0.030). Bootstrapped 95% confidence intervals confirmed the stability of the predictors; the full coefficient plot is presented in Fig. 2.

### Diagnosis-specific patterns

Criterion validity differed by diagnosis: in disc herniation, ODI (AUC = 0.741) and VAS leg (0.721) best identified dissatisfied patients. In spinal stenosis, EQ-5D-5 L index showed the strongest correlation with satisfaction (rho = − 0.440, *p* < 0.001), though ODI remained the best discriminator overall (AUC = 0.643).

## Discussion

### Principal findings

This study demonstrates that after lumbar degenerative spine surgery, pain, functional disability, and health-related quality of life recover at fundamentally different rates within the first postoperative month. This temporal asynchrony produces a multi-dimensional response discordance in which each PROM identifies a different subset of patients as treatment responders at the same timepoint. Inter-instrument agreement on MCID-based responder status was only slight to fair (κ = 0.19–0.29), nearly half of patients fell into an inter-instrument disagreement zone, and four in ten patients showed the characteristic Type A discordance pattern: pain and quality of life improved while function lagged behind. The reverse pattern did not occur, implying a hierarchical recovery progression in which sensory pain relief precedes meaningful gains in physical mobility [10, 11]. Most importantly, dissatisfaction at 1 month was independently driven by residual functional and mobility limitation and by self-reported anxiety/depression burden — not by residual pain — suggesting that early postoperative care should shift from a pain-centric paradigm toward targeted functional rehabilitation and psychological screening.

### Comparison with prior literature

Our findings extend and refine the observations of Wertli et al. [2], who reported κ = 0.24–0.50 for inter-instrument MID agreement in 466 stenosis patients at 6 months. We confirm their conclusion that instrument choice substantially affects outcome classification and add three new dimensions: a systematic discordance taxonomy (Type A/B), the finding that discordance is exclusively unidirectional at 1 month, and the identification of mobility and depression as the specific residual dimensions driving dissatisfaction. The higher disagreement in our study (κ = 0.19–0.29 vs. 0.24–0.50) is expected at an earlier timepoint when recovery trajectories across domains are most divergent.

Mannion et al. [3] reported κ = 0.56 for ODI–COMI agreement at 12 months, again suggesting that inter-instrument agreement improves as recovery progresses and outcome domains converge. This temporal gradient of concordance — from slight at 1 month to moderate at 6 months to good at 12 months — is consistent with our interpretation that the observed discordance represents differential recovery kinetics rather than a permanent measurement artefact. Several trajectory-modelling studies corroborate this interpretation, reporting that different domains may reach MCID at different timepoints across the first postoperative year [23–26]. In particular pain measures frequently show early improvement after decompression, whereas functional indices lag behind, contributing to the asymmetric discordance pattern we observed [23, 27]. Kowalski et al. [26] have highlighted that physical functioning is multidimensional and necessitates evaluation with measures beyond PROMs alone, further supporting the view that function and pain represent distinct constructs with independent recovery timelines.

The finding that residual pain does not independently predict dissatisfaction once mobility and depression are accounted for aligns with the growing recognition that psychological distress and functional limitation are central to patient experience after spine surgery [12, 13]. Preoperative and postoperative mental health states have been shown to influence satisfaction and recovery trajectories independently of pain intensity [28–32], and depressive symptoms may mediate dissatisfaction even when pain has substantially improved [30, 31, 33]. Yamamoto et al. [34] demonstrated that preoperative anxiety (HADS score ≥ median) was a significant predictor of dissatisfaction after lumbar decompression for spinal stenosis (OR = 3.95), while pain catastrophising and fear-avoidance beliefs were not, emphasising the primacy of mood-related factors over pain processing in determining satisfaction. Our results provide a mechanistic rationale for this observation through the lens of the Type A discordance pattern: pain-sensitive instruments detect early improvement from neural decompression, while function-sensitive instruments capture the slower process of musculoskeletal reconditioning. Across decompression cohorts, MCID attainment and patient satisfaction can diverge substantially, reflecting these multi-domain recovery patterns and unmet patient expectations [25, 35].

### Strengths and limitations

Strengths of this study include its prospective design within the context of an ongoing structured cohort study (PROGRES), the simultaneous assessment of four PROM families covering complementary outcome domains, the use of the Polish EQ-5D-5 L value set (Golicki et al. [15]) appropriate for the study population, externally-derived MCID thresholds — corroborated by the large CSORN registry data [20] — avoiding anchor circularity, and the inclusion of both major lumbar degenerative pathologies enabling direct comparison. Reporting of supplementary agreement statistics (Ppos, Pneg, prevalence index) alongside κ addresses the known prevalence paradox of the kappa statistic [21].

Several limitations must be acknowledged. First, the 1-month follow-up captures an early recovery snapshot rather than the definitive surgical outcome. The observed discordance likely reflects — at least in part — the natural temporal asymmetry between immediate pain relief from decompression and slower functional reconditioning; whether it persists at 3 or 12 months remains a critical question for future research. Multiple trajectory studies confirm that pain, function, and quality of life follow asynchronous courses in the early postoperative period [23–26], supporting the view that a single PROM at 1 month is insufficient to capture the full recovery landscape. Second, the sample size (*n* = 134; 31 dissatisfied) limits the regression analysis to exploratory status; external validation in a larger cohort is warranted. Third, lost-to-follow-up patients (15.2%) had marginally higher baseline ODI, potentially introducing attrition bias toward less disabled patients. Fourth, complications were not systematically captured with a standardised adverse-event protocol, and we cannot exclude the possibility that unreported minor events contributed to dissatisfaction. Fifth, the inter-instrument kappa values should be interpreted with the awareness that VAS, ODI, and EQ-5D-5 L measure different constructs by design; low agreement between conceptually distinct instruments is expected from a measurement theory perspective (Campbell and Fiske multitrait–multimethod framework [36]). The clinical relevance of our findings lies not in the kappa values per se but in the practical consequences for patient classification and care decisions. The deliberate movement restriction commonly observed during the initial six to eight weeks post-surgery may partly reflect patient compliance with postoperative precautions rather than a genuine spine-related mobility deficit [37], which should be considered when interpreting early ODI scores. Finally, mental-health status was captured only by the single self-reported EQ-5D-5 L anxiety/depression item rather than a validated psychiatric instrument; this item reflects self-reported anxiety/depression burden and should not be equated with a formal diagnosis.

### Implications for clinical practice

These findings carry three direct implications for early postoperative care. First, relying on a single PROM at the 1-month visit provides an incomplete and potentially misleading picture of recovery: a patient who is a “responder” on VAS may not be a responder on ODI, and vice versa. Multi-dimensional assessment — covering at minimum pain, function, and quality of life — is necessary to identify the 47% of patients who fall into the inter-instrument disagreement zone. Instrument selection should be diagnosis-informed: ODI and VAS leg for disc herniation, EQ-5D-5 L for spinal stenosis.

Second, since baseline PROM scores do not distinguish patients who will become dissatisfied, prospective identification of at-risk patients requires assessment at the 1-month visit rather than reliance on preoperative profiles. Screening for mobility limitation and depression/anxiety at this timepoint should be considered, given their independent predictive value. Implementing multi-domain modelling is critical because singular instruments such as the ODI may fail to explain up to 50% of the variability observed in broader health domains during the recovery period [38].

Third, for the 39.6% of patients who show Type A discordance (pain improved, function not), additional analgesia is unlikely to improve satisfaction. Hambrecht et al. [39] recently demonstrated that disaggregated ODI subsections for walking (AUC = 0.83) and personal care (AUC = 0.82) were the strongest predictors of patient satisfaction after lumbar surgery, corroborating our finding that mobility — not pain intensity — is the primary driver of patient satisfaction at the individual domain level. Early rehabilitation should therefore emphasise rapid, targeted improvements in mobility and functional activities of daily living — gait training, transfers, core stability, and graduated activity programmes — consistent with the Type A discordance pattern in which functional rehabilitation should be foregrounded in early care [23–26]. The association between depression/anxiety and dissatisfaction further supports embedding short, scalable mood screening (e.g., PHQ-9, GAD-7, or HADS) with timely mental health referral into the early postoperative pathway [28–30, 33, 34]. Such integrated behavioural health strategies can mitigate mood-related dissatisfaction and support engagement in functional rehabilitation.

## Conclusions

At 1 month after lumbar degenerative spine surgery, pain and quality of life recover substantially faster than function, creating a multi-dimensional response discordance that affects four in ten patients. Different PROMs agree on responder status in fewer than half of patients (κ = 0.19–0.29). Dissatisfaction is driven by residual functional and mobility limitation and by self-reported anxiety/depression burden, not by residual pain. The 1-month visit provides a critical window for identifying patients who require targeted functional rehabilitation and psychological support. Multi-dimensional PROM assessment at this timepoint, with diagnosis-specific instrument selection, may help identify patients who would benefit from targeted functional rehabilitation and psychological support, pending confirmation at longer follow-up.

## Supplementary Information

Supplementary Material 1.

## Data Availability

The datasets generated and analysed during the current study are not publicly available due to patient privacy considerations and institutional data governance requirements. Anonymised data supporting the conclusions of this article are available from the corresponding author on reasonable request, subject to approval by the Bioethics Committee of the Lower Silesian Medical Chamber.

## Abbreviations

Abbreviation: Full term
AUC: Area under the receiver operating characteristic curve
BMI: Body mass index
COMI: Core Outcome Measures Index
CONSORT: Consolidated Standards of Reporting Trials
CSORN: Canadian Spine Outcomes and Research Network
EPV: Events per variable
EQ-5D-5L: EuroQol five-dimension five-level questionnaire
GAD-7: Generalised Anxiety Disorder 7-item scale
HADS: Hospital Anxiety and Depression Scale
MCID: Minimal clinically important difference
MCS: Mental Component Summary
MID: Minimal important difference
MRI: Magnetic resonance imaging
N2QOD: National Neurosurgery Quality and Outcomes Database
ODI: Oswestry Disability Index
OR: Odds ratio
PHQ-9: Patient Health Questionnaire 9-item scale
Pneg: Specific negative agreement
Ppos: Specific positive agreement
PROGRES: Prospective observational study of lumbar degenerative spine disease (Evaluation of the natural course of lumbar degenerative spine disease and factors influencing its progression, with an analysis of the impact of surgical intervention as a modifying factor in the long-term course of the disease)
PROM: Patient-reported outcome measure
ROC: Receiver operating characteristic
SD: Standard deviation
SPORT: Spine Patient Outcomes Research Trial
SSSQ: Swiss Spinal Stenosis Questionnaire
STROBE: Strengthening the Reporting of Observational Studies in Epidemiology
TLIF: Transforaminal lumbar interbody fusion
VAS: Visual Analogue Scale
κ: Cohen’s kappa coefficient
