# Supplementary material for: Is pain relief enough? Early response discordance after lumbar decompressive surgery: a prospective cohort study
Source: BMC Musculoskelet Disord. 2026 Jul 10;27:837. doi: 10.1186/s12891-026-10195-3 (PMC13644135; doi:10.1186/s12891-026-10195-3)
Supplement: Supplementary file 1 [file 12891_2026_10195_MOESM1_ESM.docx]

# STROBE Checklist — Cohort Study

**Strengthening the Reporting of Observational Studies in Epidemiology**

*von Elm E, Altman DG, Egger M, et al. The Strengthening the Reporting of Observational Studies in Epidemiology (STROBE) statement: guidelines for reporting observational studies. Lancet 2007;370:1453–1457.*

**Manuscript Details**

**Title:** *Is Pain Relief Enough? Early Response Discordance After Lumbar Decompressive Surgery*

**Authors:** Tomasz Szczepański, Marta Koźba-Gosztyła, Anastazija Krzemińska, Grzegorz Miekisiak, Bogdan Czapiga

**Journal:** *BMC Musculoskeletal Disorders*

**Study design:** Prospective observational cohort study (PROGRES)

**Ethics approval:** Bioethics Committee of the Lower Silesian Medical Chamber, No. 09/DOBD/2025 (9 April 2025)

**Date completed:** March 2026

**Legend**

**✓** Criterion fully addressed in the manuscript

**N/A** Not applicable to this study design

**✓ / N/A** Partially applicable (see notes in Location column)

| **Item No.** | **Section / Topic** | **Recommendation** | **Status** | **Location in Manuscript** |
| --- | --- | --- | --- | --- |
| **TITLE AND ABSTRACT** | | | | |
| **1** | Title and abstract | (a) Indicate the study's design with a commonly used term in the title or the abstract  (b) Provide in the abstract an informative and balanced summary of what was done and what was found | **✓** | *Title page; Abstract (Background, Methods, Results, Conclusions)* |
| **INTRODUCTION** | | | | |
| **2** | Background / rationale | Explain the scientific background and rationale for the investigation being reported | **✓** | *Background section, paragraphs 1–3* |
| **3** | Objectives | State specific objectives, including any prespecified hypotheses | **✓** | *Background section, final paragraph (aims 1–3)* |
| **METHODS** | | | | |
| **4** | Study design | Present key elements of study design early in the paper | **✓** | *Methods: Study design and setting* |
| **5** | Setting | Describe the setting, locations, and relevant dates, including periods of recruitment, exposure, follow-up, and data collection | **✓** | *Methods: Study design and setting (Neurosurgical Dept., 4th Military Hospital, Wrocław; May–Dec 2025)* |
| **6** | Participants | (a) Cohort study — Give the eligibility criteria, and the sources and methods of selection of participants. Describe methods of follow-up  (b) Cohort study — For matched studies, give matching criteria and number of exposed and unexposed | **✓ / N/A** | *Methods: Participants (inclusion/exclusion criteria; no matching — N/A for 6b)* |
| **7** | Variables | Clearly define all outcomes, exposures, predictors, potential confounders, and effect modifiers. Give diagnostic criteria, if applicable | **✓** | *Methods: Outcome measures; MCID thresholds* |
| **8** | Data sources / measurement | For each variable of interest, give sources of data and details of methods of assessment (measurement). Describe comparability of assessment methods if there is more than one group | **✓** | *Methods: Outcome measures (VAS, ODI, EQ-5D-5L, SSSQ — all validated, standardised instruments)* |
| **9** | Bias | Describe any efforts to address potential sources of bias | **✓** | *Methods: MCID thresholds (externally derived to avoid circularity); Discussion: Strengths and limitations (attrition bias)* |
| **10** | Study size | Explain how the study size was arrived at | **✓** | *Methods: Statistical analysis (EPV = 6.2 reported; consecutive enrolment); Results: Patient flow (n = 134)* |
| **11** | Quantitative variables | Explain how quantitative variables were handled in the analyses. If applicable, describe which groupings were chosen and why | **✓** | *Methods: MCID thresholds (moderate, liberal, conservative thresholds defined); Statistical analysis* |
| **12** | Statistical methods | (a) Describe all statistical methods, including those used to control for confounding  (b) Describe any methods used to examine subgroups and interactions  (c) Explain how missing data were addressed  (d) Cohort study — If applicable, explain how loss to follow-up was addressed  (e) Describe any sensitivity analyses | **✓** | *Methods: Statistical analysis*  *(a) Mann–Whitney U, chi-square, Wilcoxon, κ, ROC, multivariable linear regression with bootstrapping*  *(b) Disc herniation vs. stenosis subgroups*  *(c) Complete-case analysis; lost-to-follow-up described in Results*  *(d) n = 24 lost to follow-up characterised (age, VAS, ODI)*  *(e) Sensitivity analyses at liberal/conservative MCID thresholds* |
| **RESULTS** | | | | |
| **13** | Participants | (a) Report numbers of individuals at each stage of study (e.g. numbers potentially eligible, examined for eligibility, confirmed eligible, included in the study, completing follow-up, and analysed)  (b) Give reasons for non-participation at each stage  (c) Consider use of a flow diagram | **✓** | *Results: Patient flow (n = 161 screened → 158 operated → 134 completed); Figure 1 (flow diagram)* |
| **14** | Descriptive data | (a) Give characteristics of study participants (e.g. demographic, clinical, social) and information on exposures and potential confounders  (b) Indicate number of participants with missing data for each variable of interest  (c) Cohort study — Summarise follow-up time (e.g. average and total amount) | **✓** | *Results: Patient flow and baseline characteristics; Table 1 (baseline characteristics by diagnosis); 1-month follow-up* |
| **15** | Outcome data | Cohort study — Report numbers of outcome events or summary measures over time | **✓** | *Results: Early postoperative improvement (mean changes, Cohen's d); MCID responder rates* |
| **16** | Main results | (a) Give unadjusted estimates and, if applicable, confounder-adjusted estimates and their precision (e.g. 95% CI). Make clear which confounders were adjusted for and why they were included  (b) Report category boundaries when continuous variables were categorised  (c) If relevant, consider translating estimates of relative risk into absolute risk for a meaningful time period | **✓** | *Results: Inter-instrument agreement (κ, % agreement, Ppos/Pneg); Multivariable regression (β, p, R², bootstrapped 95% CI); Table 2* |
| **17** | Other analyses | Report other analyses done — e.g. analyses of subgroups and interactions, and sensitivity analyses | **✓** | *Results: Diagnosis-specific patterns; sensitivity analyses (liberal/conservative MCID thresholds; κ range 0.15–0.31)* |
| **DISCUSSION** | | | | |
| **18** | Key results | Summarise key results with reference to study objectives | **✓** | *Discussion: Principal findings* |
| **19** | Limitations | Discuss limitations of the study, taking into account sources of potential bias or imprecision. Discuss both direction and magnitude of any potential bias | **✓** | *Discussion: Strengths and limitations (1-month snapshot; n = 134; 15.2% attrition; no adverse-event protocol; kappa paradox; postoperative movement restriction)* |
| **20** | Interpretation | Give a cautious overall interpretation of results considering objectives, limitations, multiplicity of analyses, results from similar studies, and other relevant evidence | **✓** | *Discussion: Comparison with prior literature; Implications for clinical practice* |
| **21** | Generalisability | Discuss the generalisability (external validity) of the study results | **✓** | *Discussion: Strengths and limitations (single-centre, exploratory EPV = 6.2, external validation warranted)* |
| **OTHER INFORMATION** | | | | |
| **22** | Funding | Give the source of funding and the role of the funders for the present study and, if applicable, for the original study on which the present article is based | **✓** | *Declarations: Funding (no external funding; statutory research activity of the 4th Military Hospital, Wrocław)* |

**Notes**

This checklist was completed using the STROBE statement for cohort studies (22 items). All items are either fully addressed or not applicable to the present study design. For items involving sub-items (a/b/c), individual applicability is noted in the Location column.

*The STROBE checklist is submitted as Additional File 1 to the manuscript submitted to BMC Musculoskeletal Disorders.*

**Reference:** *von Elm E, Altman DG, Egger M, Pocock SJ, Gøtzsche PC, Vandenbroucke JP; STROBE Initiative. The Strengthening the Reporting of Observational Studies in Epidemiology (STROBE) statement: guidelines for reporting observational studies. Lancet. 2007;370(9596):1453-7. doi: 10.1016/S0140-6736(07)61602-X.*
